# The Influence of Titanium Dioxide on Silicate-Based Glasses: An Evaluation of the Mechanical and Radiation Shielding Properties

**DOI:** 10.3390/ma14123414

**Published:** 2021-06-20

**Authors:** Badriah Albarzan, Mohamed Y. Hanfi, Aljawhara H. Almuqrin, M. I. Sayyed, Haneen M. Alsafi, K. A. Mahmoud

**Affiliations:** 1 Department of Physics, College of Science, Princess Nourah bint Abdulrahman University, Riyadh 11671, Saudi Arabia; baalbarzan@pnu.edu.sa (B.A.); ahalmoqren@penu.edu.sa (A.H.A.); 2 Institute of Physics and Technology, Ural Federal University, Mira St 19, 620002 Ekaterinburg, Russia; mokhamed.khanfi@urfu.ru; 3 Department of Medical and Radiological Research, Research Sector, Nuclear Materials Authority, P.O. Box 530 El-Maadi, Cairo 520, Egypt; 4 Department of Physics, Faculty of Science, Isra University, Amman 11622, Jordan; 5 Department of Nuclear Medicine Research, Institute for Research and Medical Consultations, Imam Abdulrahman Bin Faisal University, Dammam 31441, Saudi Arabia; 6 Department of Magnetism and Magnetic Nanomaterials, Natural and Mathematical Institute, Ural Federal University, St Kuibysheva 48, 620026 Yekaterinburg, Russia; haneenalsafi.1991@gmail.com; 7 Department of Nuclear Power Plants and Renewable Energy Sources, Ural Power Engineering Institute, Ural Federal University, St. Mira 19, 620002 Yekaterinburg, Russia; 8 Department of Analytical Chemistry, Production Sector, Nuclear Materials Authority, P.O. Box 530 El-Maadi, Cairo 520, Egypt

**Keywords:** silicate glass, mechanical properties, radiation shielding, MCNP5

## Abstract

The mechanical and radiation shielding features were reported for a quaternary Na_2_O-CaO-SiO_2_-TiO_2_ glass system used in radiation protection. The fundamentals of the Makishima–Mazinize model were applied to evaluate the elastic moduli of the glass samples. The elastic moduli, dissociation energy, and packing density increased as TiO_2_ increased. The glasses’ dissociation energy increased from 62.82 to 65.33 kJ/cm^3^, while the packing factor slightly increased between 12.97 and 13.00 as the TiO_2_ content increased. The MCNP-5 code was used to evaluate the gamma-ray shielding properties. The best linear attenuation coefficient was achieved for glass samples with a TiO_2_ content of 9 mol%: the coefficient decreased from 5.20 to 0.14 cm^−1^ as the photon energy increased from 0.015 to 15 MeV.

## 1. Introduction

Attention has been drawn to radiation due to their profitable applications in nuclear medicine, agriculture, etc. [1]. However, the harm of radioactive sources limits their use. Thus, the dose rate from these radiation types should be controlled. Exposure time, source-person distance, and shielding materials are essential factors for controlling exposure [2]. The most commonly known shielding materials are glass, concrete, rock, polymers, and alloys.

The type of shielding material depends mainly on the activity of the emitted energy. The most widespread and low-cost materials for X- and gamma-ray shielding are glass, heavy bricks, painting materials, and concrete [3]. Concrete is considered one of the best materials for radiation shielding due to its reasonable cost and easy construction. Concretes containing heavy and normal aggregates have been noted as suitable shielding materials against both photons and fast neutrons due to their high hydrogenous content [4]. Despite these advantages, concrete has many drawbacks. It can be damaged by expanding aggregates, the freezing of trapped water, infiltration, and other chemical and physical processes [5].

Glasses have some promising characteristics in terms of radiation protection, especially with regards to transparency. With vitrification, glasses containing heavy metal oxides (HMO) can reduce ionizing radiation hazards and keep radioactive waste chemically stable state for a long time [6]. Therefore, glasses provide transparent, light, and low-volume materials with better shielding properties [7].

Radiation shielding glasses using borate [8,9,10], phosphate, and silicate have been developed. In many applications, silicate glasses satisfy the majority of requirements [11,12]. Commercial glasses are recommended due to the ease of fabrication and the excellent provision of visibility. The absorbance and transmission of visible light are affected mainly by glass thickness and chemical composition. Increasing glass thickness will help increase the absorption edge.

Most silicate glasses are used in windows with the addition of a small amount of alkaline oxides [13]. The high atomic number of lead enhances gamma-ray attenuation. Consequently, the addition of small amounts of Na_2_O to lead oxide-based silicate glass facilitates the melting process and produces promising attenuation properties [14].

A detailed theoretical study on the mechanical properties of silicate-based lead oxide glass is provided in the present work. Furthermore, the efficiency of the investigated samples in attenuating gamma rays is examined using the Monte simulation. 

## 2. Materials and Methods

**Figure 1:**
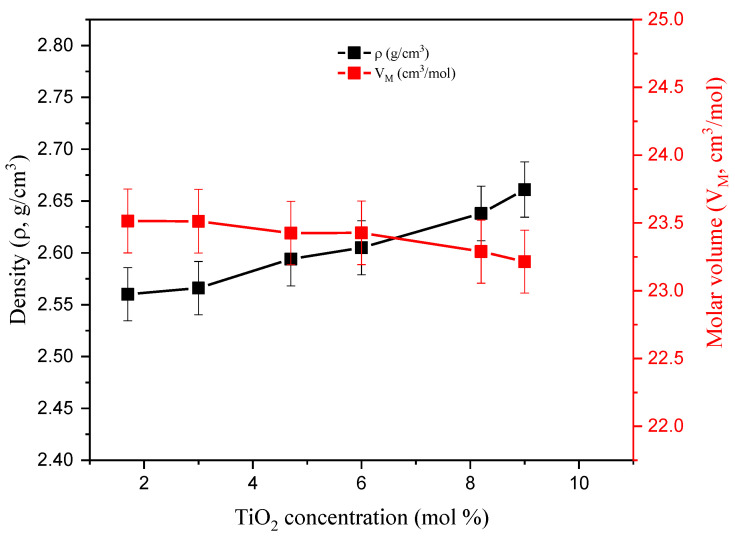
Variation of glass density and molar volume versus TiO_2_ concentration.

Six NaO_2_-CaO-SiO_2_-TiO_2_ glass samples were created. Such compounds are discussed in Limbach et al. [15]. The replacement of SiO_2_ with TiO_2_ significantly affects the physical characteristics (density *ρ*, molar volume V_M_, and molecular weight M_W_) of the quaternary Na_2_O-CaO-SiO_2_-TiO_2_ glass system. Figure 1 illustrates that density (*ρ*, g/cm^3^) increased slightly (between 2.560 and 2.661 g/cm^3^), while molar volume (V_m_, cm^3^/mol) declined from 23.51 to 23.21 cm^3^/mol. The behavior presented in Figure 1 is attributed to the partial replacement of SiO_2_ (*ρ*_SiO2_ = 2.65 g/cm^3^ and MW_SiO2_ = 60.08 g/mol) with TiO_2_ (*ρ*_TiO2_ = 4.23 g/cm^3^ and MW_SiO2_ = 79.86 g/mol).

### 2.1. Mechanical Properties

The elastic moduli (EM) and some mechanical properties were investigated. These calculations are based on the foundations provided by Makishema and Makinsize in 1973 and 1975 [16,17]. They assumed that glass atoms are found separately in the matrix and that the bonds between atoms are affected by the dissociation energy (*G_i_*) of the constituting compounds, as well as the ionic radii of metals (*R_M_*) and oxides (*R_O_*). The total dissociation energy (*G_t_*) of the investigated glass samples describes the amount of heat required to bind the metal-oxygen atoms: *G_t_* (kJ/cm^3^) = ∑*X_i_G_i_*, where *X_i_* refers to the molar fraction of the investigated samples. The packing density (*V_t_*) is related to the previously mentioned ionic radii *R_O_* and *R_M_*, where *V_t_* = (*ρ*/MW) × ∑*X_i_V_i_*: *V_i_* is the packing factor of the constituting compounds, *ρ* is the glass density, and MW is the molar weight of the glass.

Starting from *G_t_* and *V_t_*, the EM moduli (Young (*E*), bulk (*B*), shear (*K*), longitudinal (*L*)), and some mechanical properties are predicted. These mechanical properties are the Poisson ratio (*σ*), micro-hardness (*H*), the softening temperature (*T_s_*), and fractal bond connectivity (d) [18]:

(1) $$E\left(\mathrm{GPa}\right)=2V_{t}G$$

(2) $$B\left(\mathrm{GPa}\right)=1.2\;V_{t}E$$

(3) $$S\left(\mathrm{GPa}\right)=\frac{3\;EB}{\left(9B-E\right)}$$

(4) $$L\;\left(\mathrm{GPa}\right)=B+\frac{3}{4}\;S$$

(5) $$\sigma =0.5-\frac{1}{7.2}V_{t}$$

(6) $$H\left(\mathrm{GPa}\right)=\frac{\left(1-2\sigma \right)}{6\left(1+\sigma \right)}$$

(7) $$T_{s}\left(C\right)=\frac{M_{W}}{\left(\rho_{glass}\times C\right)}\times V_{s}^{2}$$

### 2.2. Shielding Properties

The gamma-ray shielding features were simulated using the Monte Carlo simulation method [19] and Phy-X/PSD software [20]. Both methods use chemical compositions and densities to evaluate shielding factors. However, they use distinct nuclear libraries, which are the most important files for extracting interaction cross-sections. MCNP-5 uses ENDF/B-VI.8 as a primary source, while the Phy-X/PSD program uses the NIST database (like XCOM software).

**Figure 2:**
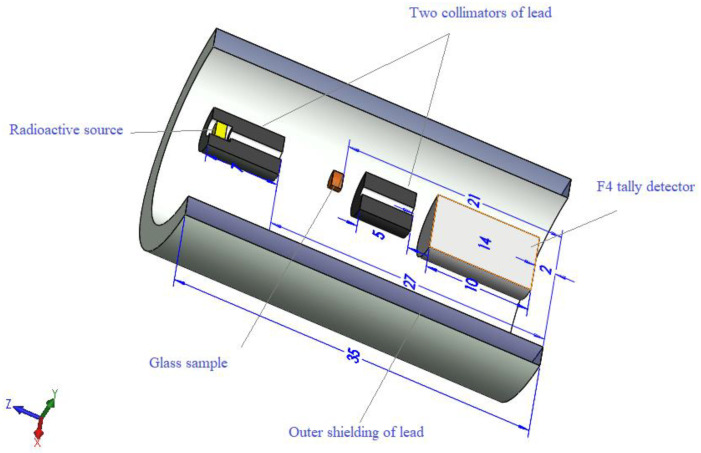
The 3D geometry described by the MCNP-5 input file.

MCNP-5 needs an input file containing geometry, cells, surfaces, sources, and detectors. Figure 2 is a 3D representation of the current input file. It shows a Pb cylinder (thickness = 5 cm, height = 35 cm, and diameter = 30 cm) and filled with dry air. This cylinder is used to isolate the equipment from external background radiation.

Inside the lead cylinder, some tools were arranged to make the simulation geometry similar to the narrow beam transmission experiment, where a source was placed in the geometry center. A lead collimator followed the source, which is then followed by the glass samples, another lead collimator, and the detector (F4 tally). The F4 tally was chosen to predict the gamma-ray average track length (ATL) over the detector cell. The dimensions of the components are illustrated in Figure 1, while the chemical composition, density, and molar volume are listed in Table 1.

The NPS card was chosen to cut off photon interactions after running at 10^8^ historical. A detailed explanation of the geometry used is presented in our previous publications [21,22].

The simulated ATL was fitted with the linear attenuation coefficient (LAC) as presented in Equations (8)–(10):

(8) $$\mu_{m}\left(\frac{{\mathrm{cm}}^{2}}{g}\right)=\frac{LAC\;\left({\mathrm{cm}}^{-1}\right)}{\rho }$$

(9) $$HVL\;\left(\mathrm{cm}\right)=\frac{\ln\left(2\right)}{LAC}$$

(10) $$MFP\;\left(\mathrm{cm}\right)=\frac{1}{LAC}$$

## 3. Results and Discussion

### 3.1. Mechanical Properties

**Figure 3:**
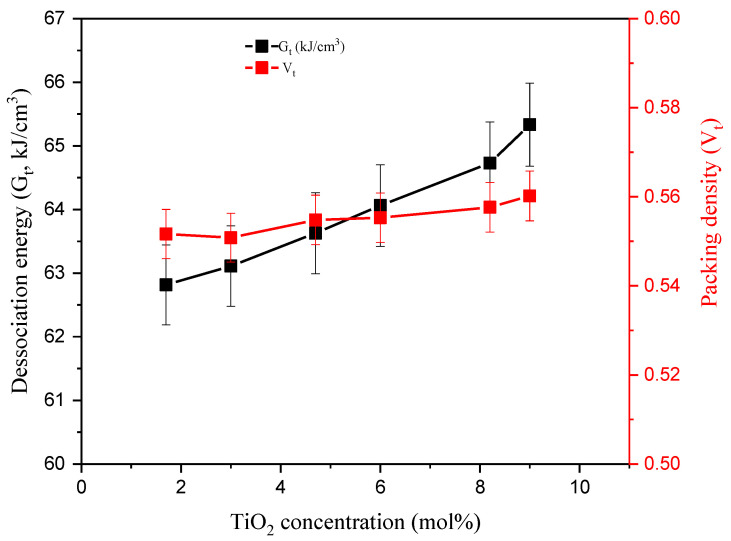
Variation of the dissociation energy (G_t_) and the packing density (*V_t_*) versus TiO_2_ concentration.

Besides the enthalpy (heat of formation) of the compound and the ionic radii values, the previously stated physical parameters were applied to calculate elastic moduli (EM) based on the assumptions of the M-M model [16,17]. The *G_t_* and *V_t_* of the glasses are calculated (Figure 3). Both *G_t_* and *V_t_* increased as SiO_2_ was replaced with TiO_2_. G_t_ is a measure of the heat of formation required to bind the glass atoms. This increased from 62.82 to 65.33 kJ/cm^3^ as the TiO_2_ concentration increased to 1.7 and 9 mol%, respectively. This is attributed to the higher bond dissociation energy of TiO_2_ (*G_i_* = 101.2 kJ/cm^3^) compared to SiO_2_ (*G_i_* = 68 kJ/cm^3^) [23]. Moreover, *V_t_* depends mainly on the ionic radii of the constituting compounds. The *V_t_* values increased slightly from 0.55 to 0.56 due to the replacement of Si atoms with an ionic radius of R_Si_ = 8.58 Å with Ti atoms (R_Ti_ = 14 Å).

**Figure 4:**
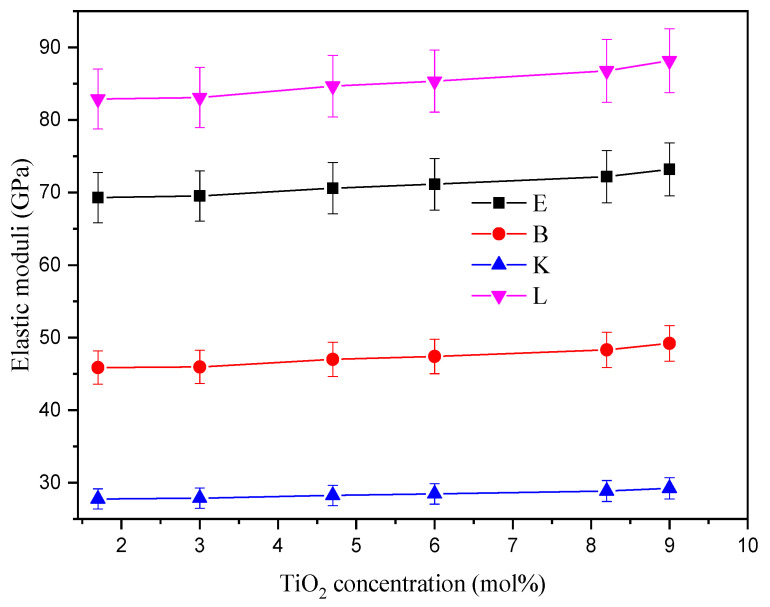
Variation of elastic moduli like the Young (*E*), bulk (*B*), shear (*K*), and longitudinal (*L*) properties as a function of TiO_2_ concentration.

EM like the Young (*E*), bulk (*B*), shear (*K*), and longitudinal (*L*) properties were also calculated based on the predicted values of *G_t_* and *V_t_*. Figure 4 illustrates that *E*, *B*, *S*, and *L* increased as TiO_2_ content increased. *E* increased from 69.30 to 73.20 GPa as the substitution of Si_2_O with Ti_2_O increased from 1.7 to 9 mol%, respectively. The other moduli followed the trend of *E*. The *B*, *K*, and *L* moduli changed between 45.88–49.21 GPa, 27.76–29.23 GPa, and 82.89–88.18 GPa, respectively. The increase of *E* is due to the increase in *V_t_* as a result of the substitution of Si-O bonds with Ti-O bonds. The calculations of *B*, *K*, and *L* are based on the E modulus (Equations (1)–(4)). Thus, they follow the same trend.

**Figure 5:**
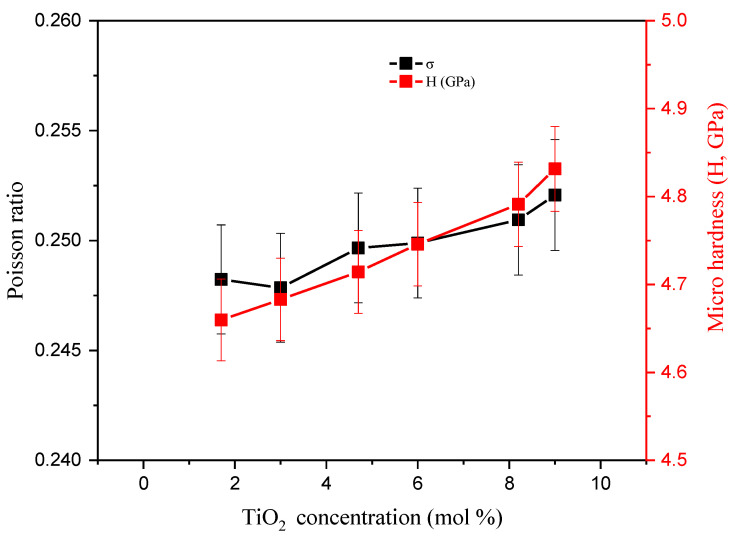
Dependence of the Poisson ratio and microhardness on TiO_2_ concentration.

Hardness is considered one of the most important mechanical properties for shielding materials. In the current study, microhardness (H, GPa) was introduced to define the hardness of these micro-scale materials. In the microhardness tests, the loads were lighter than 1 kg (less than 10 Newtons). Figure 5 shows the change in the microhardness of the glass samples when the TiO_2_ concentration increases from 1.7 to 9 mol%. The H values increased gradually with the TiO_2_ concentration, which is related to the replacement of Si-O bonds with relatively strong bonds (i.e., Ti-O). H has values of 4.66, 4.68, 4.71, 4.75, 4.79, and 4.83 GPa for glass samples with TiO_2_ content of 1.7, 3, 4.7, 6, 8.2, and 9 mol%, respectively. The Poisson ratio (*σ*) is also a measure for expanding the investigated glasses perpendicular to the compression direction. This is illustrated in Figure 5, where it follows the behavior of H. The *σ* values increase from 0.248 to 0.252 with the partial replacement of SiO_2_ with TiO_2_.

Declining temperatures were predicted for the investigated glass samples (Table 2). The temperature (*T_s_*) slightly increased as the TiO_2_ concentration increased. Samples S1, S2, S3, S4, S5, and S have values of 502.54, 503.09, 502.05, 504.44, 502.05, and 502.59 K, respectively. The slight variation is related to the replacement of SiO_2_ (with a melting point of 1710 °C) with TiO_2_ (melting point 1843 °C). Moreover, the stronger Si-O interatomic bond (field strength (F) = 1.28 Å^−2^) is substituted with the weaker Ti-O interatomic bond (F = 1.54 Å^−2^). As such, the increase in the thermal expansion coefficient leads to an increase in the T_g_ values. Concerning the T_g_ values, Takshahi et al. [24], Scannell et al. [25], and Limbach et al. [15] reported a small variation in the Tg values of xNa_2_O-yTiO_2_-(100-x-y) SiO_2_ glass system due to the substitution of SiO_2_ by TiO_2_. However, no such variation was observed by Villegas et al. [26]. These experimental studies are in agreement with our calculations.

Fractal bond conductivity (d) was close to 2 for all glass samples. This means that all the samples have a 2D layer structure network, as reported elsewhere [27,28].

Based on the EM moduli (*K* and *L*), both shear velocity (ν_s_) and longitudinal velocity (ν_l_) were predicted and listed in Table 2. Both ν_l_ and ν_s_ increase as TiO_2_ increases due to the increase in *K* and *L*. ν_l_ varied from 5690.29 to 5756.59 m/s, while νs increased from 3293.03 to 3314.36 m/s as TiO_2_ increased from 1.7 to 9 mol%.

### 3.2. Shielding Properties

**Figure 6:**
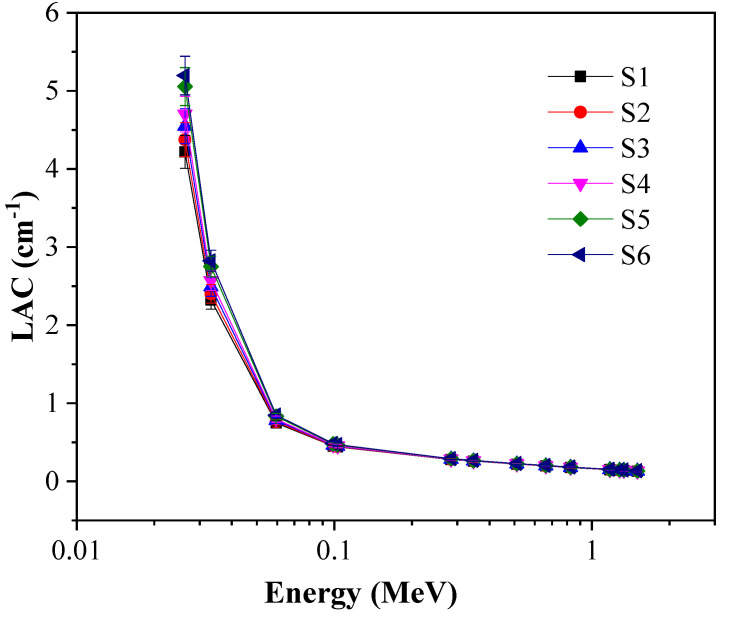
Change in LAC against incoming gamma-ray energy.

Figure 6 exhibits the LAC against the energy of the incident photons and the concentration of TiO_2_ from 1.7 to 9 mol%. First, let us consider the effect of the energy of incoming photons on LAC values. As shown in Figure 6, at 0.0263 MeV, the highest LAC values are present: photoelectric effect (PE) interactions are predominant, and the PE cross-section is directly proportional to incoming photon energy (E^−3.5^) [29]. As an example, for S1, the LAC values decrease from 4.2 cm^−1^ to 0.13 cm^−1^ at 0.0263 and 1.5 MeV, respectively. The rapid wane of the LAC values was detected with continuous increase of incoming photon energy. The previous trend in LAC can be demonstrated according to the Compton Scattering (CS) interactions prevalent and its cross-section impacted with the effective atomic number Z_eff_ (*σ*_CS_ α Z_eff_). The LAC values can be disregarded at the high energy range, where the studied glasses provide ineffective shielding. As can be seen at 0.0263 MeV, the LAC values are 4.2 and 5.19 cm^−1^ for the glasses with 1.7 mol% (S1) and 9 mol% (S6) of TiO_2_ content, respectively. As opposed to the high photon energy (1.5 MeV), the LAC values are 0.13 and 0.14 cm^−1^ for the glasses with TiO_2_ content of 1.7 mol% for S1 and 9 mol%. At constant photon energy values, the LAC values change with the addition of TiO_2_. MW increased from 60.2 to 61.77 g/mol for S1 and S6, respectively, while the density increased from 2.56 to 2.661 g/cm^3^. Thus, the LAC values rise as TiO_2_ increases from 1.7 to 9 mol%.

The theoretical mass attenuation coefficient (MAC) was computed using the Phy-X database: the values were compared with the simulated MAC and are listed in Table 3.

The difference (Δ%) between the theoretical and simulated MAC values is calculated by Equation (11). The difference is lower than 2% for all S glasses.

(11) $$\Delta \;\left(\%\right)=\frac{\left[{\left(\mu_{m}\right)}_{mcnp}-{\left(\mu_{m}\right)}_{xcom}\right]}{{\left(\mu_{m}\right)}_{mcnp}}\;\times 100$$

**Figure 7:**
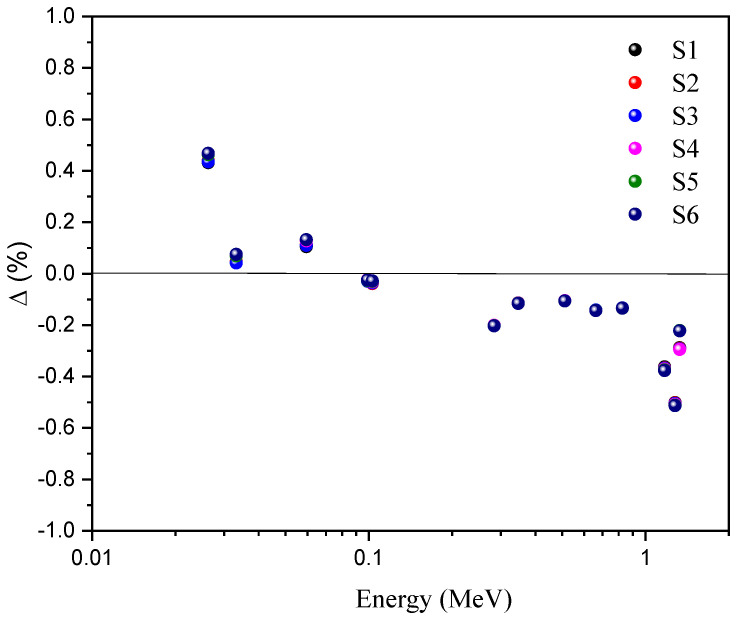
Ratios of MAC values computed by Phy-X against those simulated with the MCNP code.

The difference ranges over −0.288–0.432% for S1, −0.290–0.439% for S2, −0.292–0.435% for S3, −0.294–0.463% for S4, −0.221–0.463 and −0.222–0.468% for S6. Figure 7 illustrates the inconsistent comparison of the theoretically computed data with the simulated coefficients. From this, it is plain that gaps are not observed between the Phy-X and MCNP5 data. In general, the computed results from MAC via Phy-X are slightly lower than the simulated results using MCNP5. This may have been caused by the fact that the Phy-X code does not include the effects of the atomic wave mechanism on molecular bonding, which may lead to a decrease in MAC in such compounds. The smallness of the relative deviations can be ascribed to the precision of the code’s physics models [30,31].

**Figure 8:**
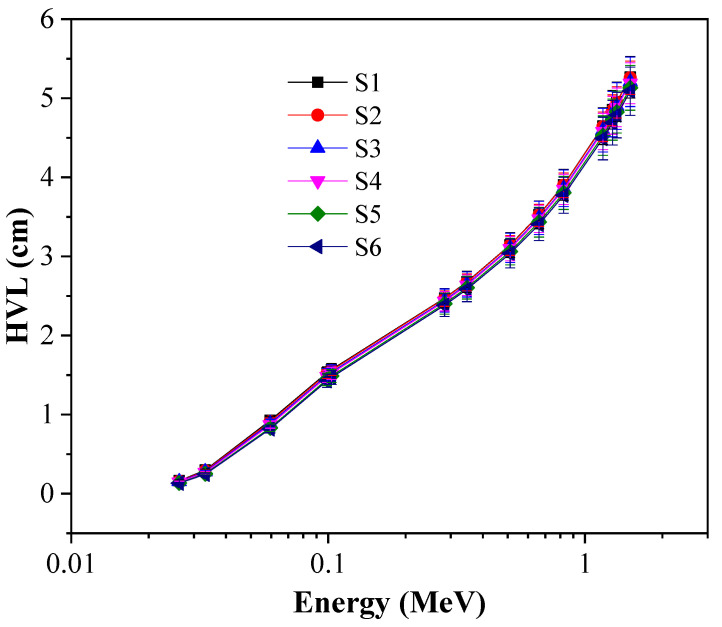
HVL of glasses as a function of energy.

Unlike LAC, the simulated HVL values increase as the incoming gamma photon energy increase (Figure 8). These values demonstrate the ability of the investigated glasses to reduce the incoming gamma photon energy by half. The simulated HVL reached maximum values of 5.2 and 5.08 cm at high gamma energy (1.5 MeV) for S1 and S6. This means that the glasses with low simulated HVL value can be applied as radiation shielding. S6 glasses have low simulated HVL values at the applied range of incoming gamma photon energy (0.0263–1.5 MeV).

**Figure 9:**
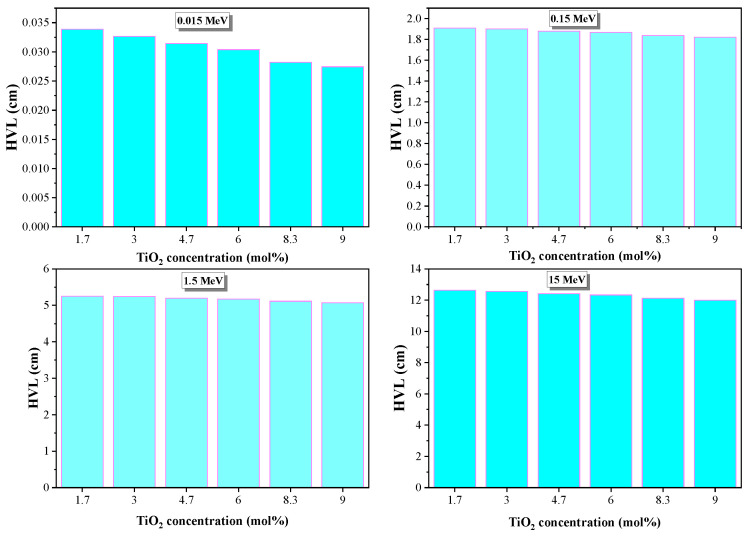
Changes in HVL in the glasses at stationary gamma photon energy (0.015, 0.15, 1.5, and 15 MeV).

Figure 9 depicts how TiO_2_ content influenced the HVL values. The computer program Phy-x/PD was used to compute the theoretical HVL values at varying incoming photon energies (0.015–15 MeV). As can be noted, the HVL values decreased as TiO_2_ content increased from 1.7 to 9 mol % at a stationary photon energy. At all selected photon energies, the lowest HVL values are achieved by S6, while the highest are observed for S1.

**Figure 10:**
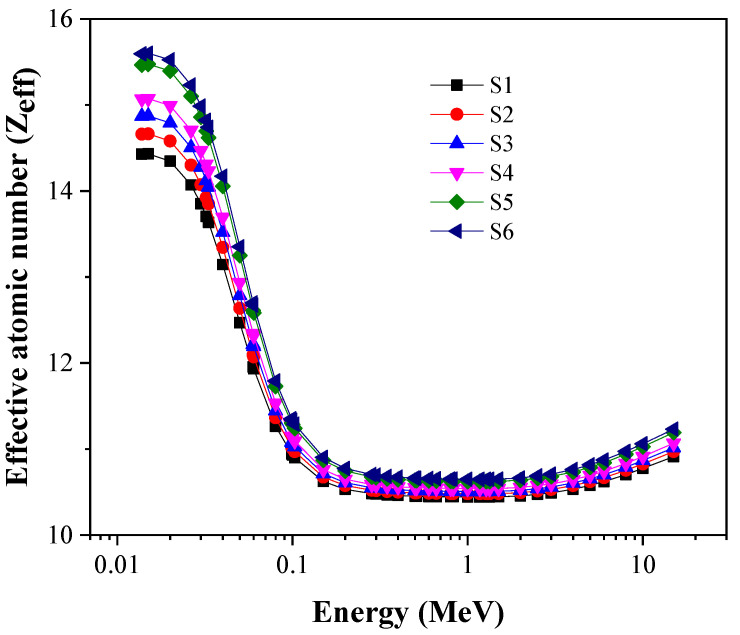
Change in Z_eff_ as a function of gamma-photon energy.

**Figure 11:**
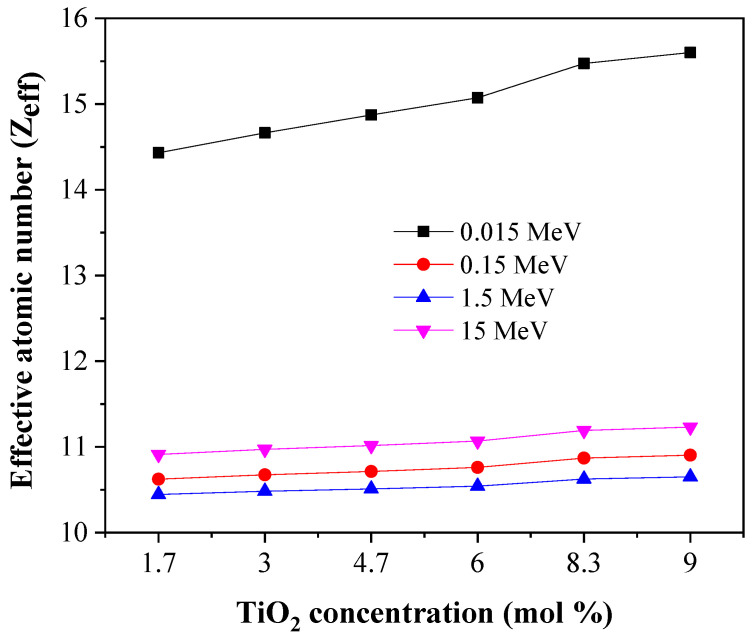
Change in Z_eff_ at a fix four photon energies.

**Figure 12:**
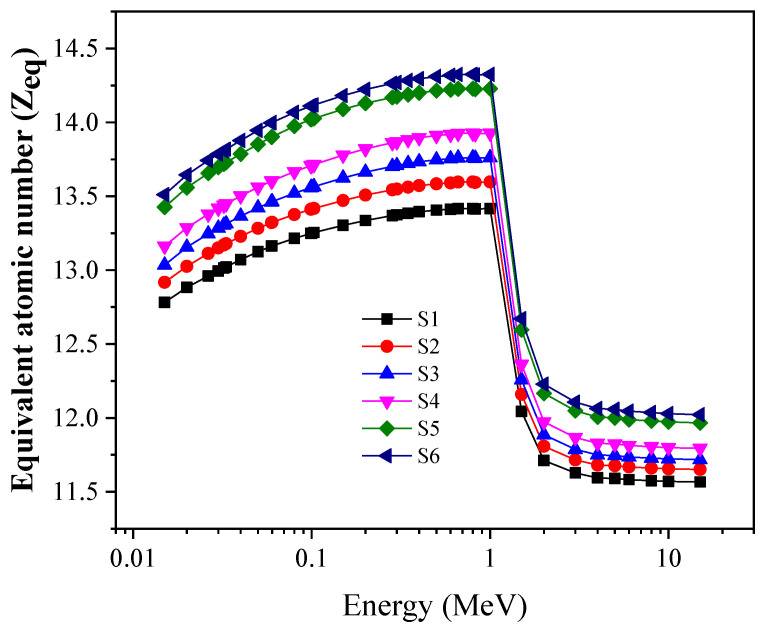
Change in equivalent atomic number (Z_eq_) against incoming photon energy.

**Figure 13:**
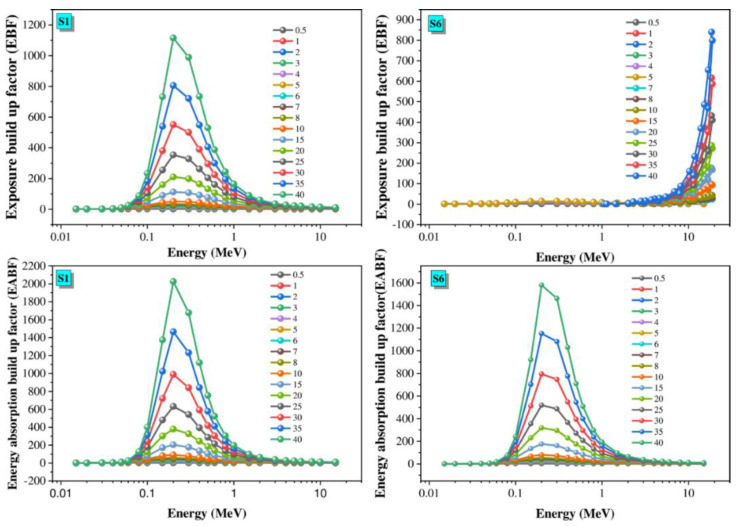
Dependence of EBF and EABF on the radiation energy.

Further, the effective atomic number (Z_eff_), the equivalent atomic number (Z_eq_), the exposure build-up factor (EBF), and the energy absorption build-up factor (EABF) were computed through the Phy-x/PD. The data are presented in Figure 10, Figure 11, Figure 12 and Figure 13 and are discussed in the following.

First, the effective atomic number (Z_eff_) values have been observed so that we can study the ability of the glasses to be used as shielding against gamma radiation. Figure 10 and Figure 11 show the Z_eff_ profile for the tested glasses. In Figure 10, the Z_eff_ values are affected by the energy of the incoming photons. In the selected energy range, three main interactions are detected. The first interaction is PE, which possesses a photon energy range of 0.015–0.1 MeV: this changed with Z^4^ and the maximum Z_eff_ values. Clearly, the Z_eff_ values drop quickly as photon energy increases. When photon energy increases above 0.1 MeV, the CS interaction is predominant: Z_eff_ gradually decreases. At photon energy levels greater than several MeV, Z_eff_ starts to increase. This change is attributed to the pair production (PP) interaction, which is predominant at high gamma-ray energy intervals. The PP interaction cross-section directly changes with Z^2^ [32].

Figure 10 and Figure 11 show that the Z_eff_ values decline when TiO_2_ increases from 1.7 to 9 mol %. Thus, the insertion of TiO_2_ enhances shielding effectiveness significantly. S6 is the most promising sample for radiation protection compared to the others.

The second factor is the Z_eq_, which is calculated based on the MACCS values and presented in Figure 12. The values of Z_eq_ increased as the energy increased from 0.015 to 1 MeV. The equivalent atomic number reaches the highest values in the CS region, the intermediate photon energy range. At the same time, the minimum values of Z_eq_ are achieved in the high-energy region, where the PP interactions occur. The maximum values of Z_eq_ increase from 12.78 to 13.51 for S1 and S6, respectively, while the minimum values increase from 11.56 to 12.02 for S1 and S6, respectively.

EBF and EABF are two factors used to determine the total flux of gamma radiation. The variation of both parameters with the incoming energy for S1 and S6 is presented in Figure 13. Moreover, Table 4 illustrates the G-P fitting factors of the EBF and EABF values for S6, which is optimized at gamma energies ranging from 0.015 to 15 MeV.

Figure 13 displays the change in the EBF and EABF values as incoming photon energy increases up to 15 MeV. The minimum EBF and EABF are realized in the low-energy region. This is associated with the photoelectric process, which means all incident gamma photons will pass through the glass. Otherwise, the values of EBF and EABF increase as the incoming photon energy increases in the middle photon energy range. This is due to the track of multiplied scattering photons from the Compton process. This clarifies the number of incident gamma photons interacting and penetrating the glass material: the remaining photons are scattered, inducing more interactions. Therefore, multiple photons accumulate inside the glass material. Finally, in the high energy range, the EBF and EABF values reach their maximum values, which is associated with the PP process [33,34].

**Figure 14:**
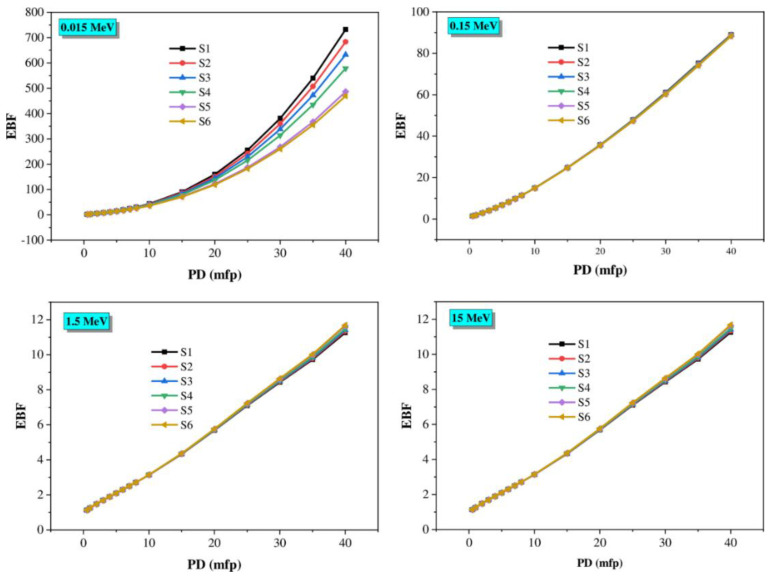
EBF changes against the penetration depth of the glasses at various gamma photon energies (0.015, 0.15, 1.5, and 15 MeV).

**Figure 15:**
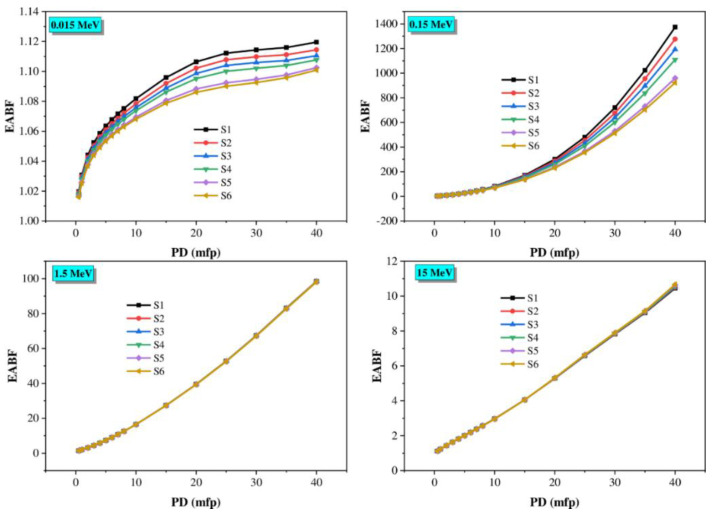
EABF changes against the penetration depth of the glasses at various gamma photon energies (0.015, 0.15, 1.5, and 15 MeV).

Moreover, the buildup factors were affected by penetration depth (PD, mfp), which varied from 0.5 to 40 mfp at the four selected photon energies. The buildup factors depend on the composition of the samples (Figure 14 and Figure 15). The correlation between EBF, EABF, and PD values is clearly shown in Figure 14 and Figure 15. The glasses with the lowest PD value have low EBF and EABF values because the incident gamma photons spend only a short time inside the material. At the same time, the highest values are due to the long period that photons spend within the glass. Furthermore, TiO_2_ in the glasses leads to an increase in the buildup factors.

## 4. Conclusions

In this study, we have considered the effect of replacing SiO_2_ with TiO_2_ on the mechanical and radiation shielding properties of a quaternary glass system consisting of Na_2_O, CaO, SiO_2_, and TiO_2_. The partial replacement of SiO_2_ showed enhanced elastic moduli, and radiation shielding of the glass system. The Young module increased from 69.30 to 73.20 GPa as TiO_2_ content increased from 1.7 to 9 mol%. The bulk, shear, and longitudinal moduli followed the same trend. The microhardness increased from 4.66 to 4.83 GPa as the SiO_2_ substitution ratio increased. The radiation shielding properties were evaluated using MCNP-5 and the Phy-X/PSD program between 0.015 and 15 MeV, which covers the entire experimental energy range. Both Phy-X/PSD and MCNP-5 results are in good agreement. The highest LAC value was detected at 0.015 MeV: it increased from 4.22 to 5.20 cm^−1^ as the TiO_2_ concentration increased from 1.7 to 9 mol%. The EBF and EABF calculations showed that the replacement of SiO_2_ with TiO_2_ reduces photon accumulation. The advantages of the present glass samples means they offer good low-cost shielding for the low- and mid-energy regions.

## Figures and Tables

**Table 1:** Chemical composition of the glass samples.

Samples	Chemical Composition (mol%)				Density (g/cm^3^)	MW (g/mol)	V_m_ (cm^3^/mol)
	Na_2_O	CaO	SiO_2_	TiO_2_			
S1	14.6	14.0	69.8	1.7	2.6	60.2	23.5
S2	14.6	14.0	68.3	3.0	2.6	60.3	23.5
S3	15.0	13.3	67.0	4.7	2.6	60.8	23.4
S4	15.0	13.2	65.8	6.0	2.6	61.0	23.4
S5	15.1	13.9	62.8	8.3	2.6	61.4	23.3
S6	15.0	13.8	61.3	9.0	2.7	61.8	23.2

**Table 2:** Mechanical properties of the investigated glass samples.

Sample	*V_i_*	ν_l_ (m/s)	ν_s_ (m/s)	Softening Temperature (*T_s_*, °C)	Fractal Bond Conductivity (d)
S1	12.97	5690.29	3293.03	502.54	2.42
S2	12.95	5690.78	3294.95	503.09	2.42
S3	13.00	5713.09	3299.95	502.75	2.40
S4	13.01	5724.26	3305.41	504.44	2.40
S5	12.99	5735.65	3307.32	502.05	2.39
S6	13.00	5756.59	3314.36	502.59	2.38

**Table 3:** The mass attenuation coefficient of the investigated glass samples using MCNP5 and Phy-X program.

Energy	Mass Attenuation Coefficient (cm^2^/g)																	
(MeV)	S1			S2			S3			S4			S5			S6		
	MCNP-5	Phy-X	∆ (%)	MCNP-5	Phy-X	∆ (%)	MCNP-5	Phy-X	∆ (%)	MCNP-5	Phy-X	∆ (%)	MCNP-5	Phy-X	∆ (%)	MCNP-5	Phy-X	∆ (%)
0.0263	1.648	1.641	0.432	1.705	1.697	0.439	1.753	1.746	0.435	1.805	1.797	0.463	1.916	1.907	0.463	1.953	1.943	0.468
0.0332	0.907	0.907	0.042	0.936	0.935	0.047	0.960	0.960	0.042	0.986	0.986	0.072	1.042	1.042	0.069	1.061	1.060	0.075
0.0595	0.292	0.291	0.105	0.297	0.296	0.113	0.301	0.301	0.110	0.306	0.305	0.128	0.315	0.315	0.132	0.318	0.318	0.132
0.099	0.177	0.177	−0.029	0.178	0.178	−0.027	0.179	0.179	−0.029	0.180	0.180	−0.024	0.182	0.182	−0.026	0.182	0.182	−0.025
0.103	0.172	0.173	−0.037	0.173	0.173	−0.039	0.174	0.174	−0.036	0.175	0.175	−0.032	0.177	0.177	−0.030	0.177	0.177	−0.028
0.284	0.110	0.110	−0.201	0.109	0.110	−0.202	0.109	0.110	−0.203	0.109	0.110	−0.201	0.109	0.110	−0.202	0.109	0.110	−0.203
0.347	0.101	0.101	−0.114	0.101	0.101	−0.115	0.101	0.101	−0.115	0.101	0.101	−0.113	0.101	0.101	−0.115	0.101	0.101	−0.116
0.511	0.086	0.086	−0.105	0.086	0.086	−0.106	0.086	0.086	−0.105	0.086	0.086	−0.105	0.086	0.086	−0.106	0.086	0.086	−0.106
0.662	0.077	0.077	−0.142	0.077	0.077	−0.142	0.077	0.077	−0.141	0.077	0.077	−0.143	0.077	0.077	−0.143	0.076	0.077	−0.143
0.826	0.069	0.069	−0.134	0.069	0.069	−0.134	0.069	0.069	−0.134	0.069	0.069	−0.134	0.069	0.069	−0.134	0.069	0.069	−0.134
1.173	0.058	0.058	−0.363	0.058	0.058	−0.366	0.058	0.058	−0.368	0.058	0.058	−0.371	0.058	0.058	−0.374	0.058	0.058	−0.377
1.28	0.056	0.056	−0.501	0.056	0.056	−0.503	0.056	0.056	−0.505	0.056	0.056	−0.508	0.056	0.056	−0.512	0.056	0.056	−0.513
1.33	0.055	0.055	−0.288	0.055	0.055	−0.290	0.055	0.055	−0.292	0.054	0.055	−0.294	0.054	0.055	−0.221	0.054	0.055	−0.222

**Table 4:** G-P fitting parameters for S6 at various gamma photon energies.

*E* (MeV)	Z_eq_	EBF					EABF				
		a	b	c	d	X_k_	a	b	c	d	X_k_
0.015	13.51	0.24	1.03	0.37	−0.16	13.67	0.21	1.03	0.39	−0.12	12.58
0.02	13.65	0.19	1.06	0.41	−0.11	16.31	0.2	1.06	0.4	−0.11	16.47
0.03	13.79	0.21	1.19	0.4	−0.11	14.22	0.21	1.19	0.4	−0.12	14.33
0.04	13.88	0.19	1.4	0.47	−0.10	14.38	0.19	1.41	0.46	−0.10	14.64
0.05	13.95	0.13	1.65	0.58	−0.07	15.18	0.12	1.69	0.6	−0.06	16.73
0.06	14	0.09	1.89	0.7	−0.05	15.29	0.14	2.18	0.6	−0.08	13.56
0.08	14.07	0.06	2.39	0.83	−0.05	14.54	0.07	3.04	0.81	−0.05	13.96
0.1	14.11	0.01	2.6	1.01	−0.03	13.7	0.01	3.68	1.02	−0.03	13.76
0.15	14.18	−0.04	2.71	1.26	−0.01	10.63	−0.06	4.03	1.33	0.01	15.07
0.2	14.22	−0.06	2.66	1.36	−0.01	8.02	−0.08	3.69	1.48	0.02	14.78
0.3	14.27	−0.08	2.47	1.46	0.01	17.52	−0.10	3.12	1.57	0.03	14.37
0.4	14.3	−0.08	2.35	1.46	0.02	16.23	−0.10	2.79	1.56	0.03	14.77
0.5	14.31	−0.08	2.24	1.45	0.02	16.3	−0.09	2.58	1.53	0.03	15.01
0.6	14.32	−0.08	2.17	1.42	0.02	17.94	−0.09	2.44	1.49	0.03	14.99
0.8	14.32	−0.07	2.05	1.38	0.02	15.4	−0.08	2.24	1.42	0.03	15.12
1	14.33	−0.06	1.97	1.32	0.02	16.21	−0.07	2.12	1.36	0.02	14.99
1.5	12.67	−0.05	1.86	1.23	0.02	15.53	−0.05	1.94	1.25	0.02	14.65
2	12.23	−0.03	1.79	1.15	0.01	15.92	−0.04	1.83	1.16	0.01	14.53
3	12.11	−0.01	1.67	1.06	0	15.86	−0.01	1.7	1.06	0	14.34
4	12.06	0.01	1.6	1	−0.01	12.97	0.01	1.61	0.99	−0.01	14.35
5	12.06	0.02	1.54	0.94	−0.02	10.21	0.03	1.55	0.92	−0.03	13.09
6	12.05	0.02	1.48	0.93	−0.02	12.02	0.02	1.47	0.93	−0.03	15.4
8	12.04	0.03	1.4	0.9	−0.03	13.85	0.03	1.38	0.91	−0.02	12.04
10	12.03	0.04	1.34	0.89	−0.03	13.09	0.03	1.31	0.92	−0.03	14.56
15	12.02	0.06	1.25	0.85	−0.05	14.25	0.06	1.23	0.84	−0.05	14.16

## Data Availability

The data presented in this study are available on request from the corresponding author.
